# A Case of Malignant Priapism Secondary to Muscle Invasive Bladder Cancer and Review of Palliative Management

**DOI:** 10.1155/criu/2340917

**Published:** 2025-07-18

**Authors:** David Fenton, Simon Han, Adrianna Lee, Alexandra Hernandez Perez, Kristina Gam, Jung Woo Kwon, Piyush Agarwal, Omer Raheem

**Affiliations:** ^1^Department of Urology, University of Minnesota, Minneapolis, Minnesota, USA; ^2^Pritzker School of Medicine, University of Chicago, Chicago, Illinois, USA; ^3^Department of Surgery, Section of Urology, University of Chicago, Chicago, Illinois, USA; ^4^Department of Pathology, University of Chicago, Chicago, Illinois, USA; ^5^Department of Surgery, Section of Urology, Cleveland Clinic, Abu Dhabi, UAE

**Keywords:** corporal tunneling and decompression, malignant priapism, pain control, palliative management

## Abstract

Malignant priapism secondary to a genitourinary malignancy is a rare and late-stage oncological finding. A 72-year-old man with a past medical history of muscle-invasive bladder cancer treated with trimodal therapy presented with altered mental status, white discharge from his Foley catheter, and bilateral corporal rigidity. Initial pelvic magnetic resonance imaging demonstrated engorgement of the corporal bodies without obvious tumor invasion. The patient eventually underwent penile exploration and bilateral decompression, and a biopsy revealed high-grade urothelial carcinoma invading the corporal tissues. Despite current guidelines for priapism, we present a three-tiered approach to the management of malignant priapism.

## 1. Introduction

Malignant priapism (MP) is often a late-stage cancer finding defined by primary penile involvement of malignancy or metastasis resulting in clinical priapism [1–3]. MP is most commonly secondary to genitourinary malignancies and is the initial presentation of the cancer in 20%–50% of cases [4]. Regardless of its origin, MP is a late-stage cancer finding and is associated with poor outcomes. In our case report, we describe a case of MP from metastatic urothelial bladder cancer, which was treated with palliative pain control and corporal tunneling. Additionally, we present a three-tiered approach to management based on prior cases highlighted within the literature.

## 2. Case Presentation

A 72-year old man with a past medical history of hypertension, Type II diabetes mellitus, gastric carcinoid tumor status post endoscopic resection, and muscle invasive bladder cancer (MIBC) presented to our emergency department with fatigue and altered mental status. The patient's oncologic history was significant for extensive necrosis and muscle invasion on secondary transurethral resection of the bladder tumor (TURBT) which was complicated postoperatively by bilateral pulmonary embolisms and inferior vena cava filter placement. He had completed trimodal therapy with capecitabine and irradiation 1 month prior and had a Foley catheter for his chronic urinary retention.

**Figure 1:**
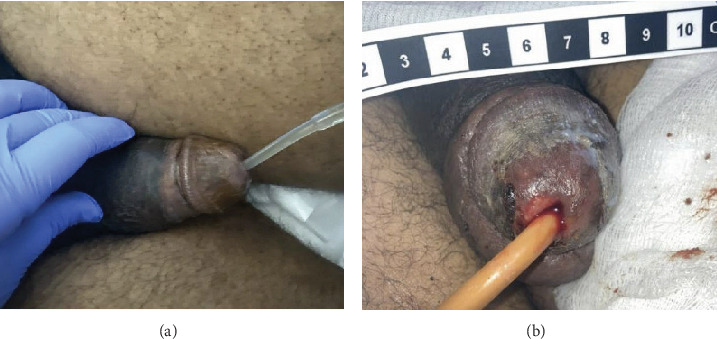
Genitourinary examination of the penis (a) before and (b) after penoscrotal decompression. No signs of necrosis were notable postoperatively.

**Figure 2:**
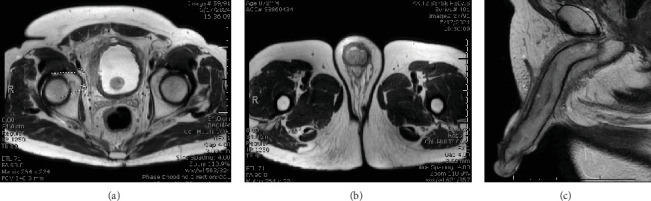
(a) Pelvic MRI highlighting the 1.6 cm right lateral bladder wall defect adjacent to the TURBT defect. (b) There was increased diffusion restriction of the R corpus cavernosum initially hypothesized to be secondary to infection or malignancy. (c) Sagittal view shows engorgement of the corporal bodies without obvious tumor invasion.

On presentation, the patient was hemodynamically stable and evaluation of his Foley catheter revealed malodorous urine with notable white discharge. Genitourinary examination demonstrated bilateral rigidity of the proximal corporal bodies, with increased rigidity on the right, and erythema surrounding the corpora and corona (Figure 1a). The remainder of his physical exam was unremarkable. Initial laboratory findings were significant for a creatinine level of 0.76 mg/dL, hemoglobin of 8.4 g/dL, a white blood cell count of 6.2 × 10^3^/*μ*L, a urine culture with mixed flora (< 10,000 CFU/mL), and an arterial blood gas with a pH of 7.41. A penile duplex performed revealed a 100% right middle versus distal dorsal artery occlusion and retrograde waveforms of the right cavernosal artery suggestive of a proximal level obstruction. Pelvic magnetic resonance imaging (MRI) revealed a 1.6-cm mass found on the right lateral wall of the bladder adjacent to his previous TURBT resection site, diffusion restriction in the right corpus cavernosum, and engorgement of the corporal bodies without obvious tumor invasion (Figures 2a, 2b, and 2c). At the time, the patient was treated empirically for a complicated UTI and was discharged with 2 weeks of antibiotics.

**Figure 3:**
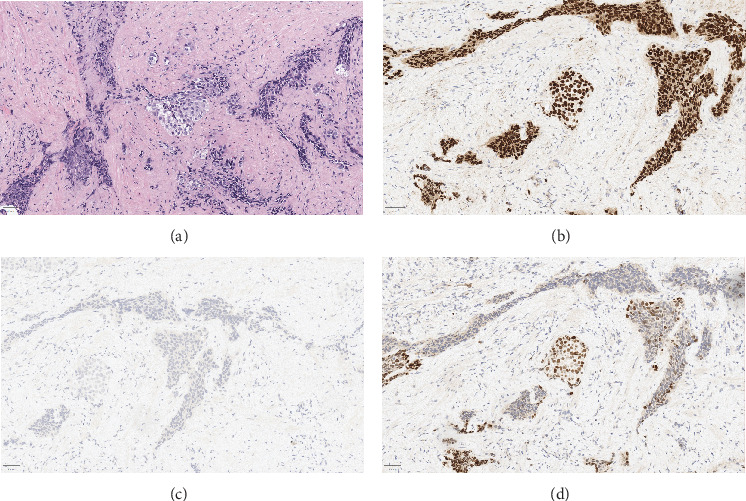
(a) Hematoxylin and eosin image of high-grade urothelial carcinoma infiltrating the corporal tissue. (b) The tumor cells are positive for urothelial markers p63, (c) negative for prostate-specific antigen, and (d) positive for GATA3 by immunohistochemistry, confirming the urothelial origin.

He later presented with increasing weakness and persistent penile pain and subsequently underwent penile exploration and bilateral penoscrotal decompression with drain placement. Bilateral proximal corporal incisions resulted in the immediate release of congested, deoxygenated blood mixed with purulent discharge, which was collected for culture. The corporal bodies were then decompressed and dilated using 9-mm Hegar dilators to relieve pressure and further drain residual blood and pus. A biopsy was taken for pathologic diagnosis. Following corporal fluid and tissue collection, the corporal bodies were closed, and a 10 French Jackson–Pratt surgical drain was placed in the left hemiscrotum to manage postoperative drainage. Blood cultures were unremarkable, but pathology revealed high-grade urothelial carcinoma infiltrating the corporal tissue bilaterally (Figures 3a, 3b, 3c, and 3d).

Postoperatively, the patient's priapism and constitutional symptoms had improved, and he remained on broad-spectrum antibiotics with plans to begin outpatient palliative chemotherapy and radiotherapy (Figure 1). Additional interventions were deferred due to poor surgical candidacy and increased concern for poor wound healing or extensive resection upon further cavernosal drainage or debridement. The patient was discharged 1 month after admission with a plan for outpatient palliative hospice care following discussions with the patient and his family about goals of care.

## 3. Discussion

MP remains an exceedingly rare and poor prognostic indicator for primary metastatic disease [1]. Current guidelines from the American Urological Association for priapism provide a framework for management techniques for MP [5]. Determining ischemic or nonischemic etiology is pertinent in the management of priapism and can be confirmed based on physical exam findings, corporal blood gas analyses, penile doppler ultrasound, and ancillary imaging (CT, MRI, or angiography). For ischemic priapism, aspiration or irrigation is performed initially either at bedside or in the OR and may be escalated to distal or proximal shunting or corporal tunneling procedures. Conservative management is generally sufficient for nonischemic priapism, and in refractory cases, selective angioembolization may be pursued.

For patients with high clinical suspicion of MP, common causes and patient's high-risk factors (hematological disorders, medications, trauma, and illicit drug activity) should be closely considered. In addition, confirmatory imaging or cytology/pathology should be pursued before initiating treatment. While delineating the mechanism of ischemic versus nonischemic is still beneficial in management, the goal of treatment is focused on palliative pain control and end-of-life care, rather than preserving erectile function [6]. Moreover, determining the origin of cancer may help guide overall management. Though rare, small locoregional disease secondary to penile squamous cell carcinoma may be curable with aggressive multimodal therapy and excision [7]. This approach may differ significantly from that used for cancers of other origin.

Following the stepwise treatment outlined for priapism provides a helpful framework in overall management but may result in inefficient or inadequate management. Based on current literature, we propose a three-tiered approach in palliative management—multimodal pain control, nonsurgical management, and surgical management. These approaches may be utilized in combination with one another and, moreover, should be determined based on patient-centered factors, such as cancer origin, prognosis, life expectancy, social determinants of health, and other significant comorbidities [6].

For poor surgical candidates, patients with significant morbidity, or terminally ill patients, palliative pain control may be an adequate option. Prior cases have demonstrated the utility of a multimodal approach with oral pain medications, patient-controlled analgesia, and penile nerve blocks (dorsal, cavernosal, and/or ring blocks). One recent case leveraged a minimally invasive ultrasound-guided dorsal neurolysis to significantly improve pain control while also reducing systemic morphine by 50% [8]. These may be repeated as necessary for sufficient pain control; however, escalating therapeutic options may be required.

Nonsurgical management consists of palliative chemotherapy and radiotherapy. Patients with hematological-based malignancies can be treated in the outpatient setting with appropriate chemotherapy based on cancer origin [6]. In treating primary lymphoma, one study found one cycle of E-CHOP preserved penile structure and function while maximizing pain control [9]. Similarly, novel radiation techniques have demonstrated favorable outcomes. One systematic review outlined favorable options for radiation intensities and fractions in addition to supporting treatment with chemotherapy or hormonal therapy. Outcomes across all reported cases were favorable, with noted pain relief, complete resolution, or decrease in tumor volume [10].

In patients who require urgent decompression or who fail pain control with other conservative options, surgical management is often considered most definitive. Following step-wise management outlined by the AUA may be pursued, initially starting with corporal aspiration then penile shunting either proximally or distally [11]. If these options fail, however, total or partial penectomies are considered the last resort but are most effective in achieving long-term symptomatic relief [2].

## 4. Conclusion

Prognoses for patients with MP remain generally poor. While current AUA guidelines for treatment of priapism provide a framework for treatment, they are not comprehensive in treating MP. The uncommon nature of MP makes finding a consensus on management difficult. Current studies recommend highlighting palliative pain management and end-of-life care.

## Data Availability

The data that support the findings of this study are available on request from the corresponding author. The data are not publicly available due to privacy or ethical restrictions.
